# Phase II, Double-Blinded, Randomized, Placebo-Controlled Clinical Trial Investigating the Efficacy of Mebendazole in the Management of Symptomatic COVID-19 Patients

**DOI:** 10.3390/ph16060799

**Published:** 2023-05-29

**Authors:** Mohamed El-Tanani, Khaled Abdul-Aziz Ahmed, Ashok K. Shakya, Wesam G. Ammari, Abdel-Elah Al-Shudifat

**Affiliations:** 1 Pharmacological and Diagnostic Research Centre (PDRC), Al-Ahliyya Amman University, Amman 19328, Jordan; 2 Institute of Cancer Therapeutics, Faculty of Life Sciences, University of Bradford, Bradford BD7 1DP, UK; 3 Department of Medical Laboratory Sciences, Faculty of Allied Medical Sciences, Al-Ahliyya Amman University, Amman 19328, Jordan; 4 Department of Internal and Family Medicine, Faculty of Medicine, The Hashemite University, Zarqa 13133, Jordan

**Keywords:** placebo-controlled clinical trial, mebendazole, COVID-19 outpatients, repurposing

## Abstract

The outbreak of the COVID-19 pandemic has spread throughout the world, affecting almost all nations and territories. The current double-blind, randomized, placebo-controlled, phase II clinical trial sought to evaluate the clinical efficacy and safety of mebendazole as an adjuvant therapy for outpatients with COVID-19. The patients were recruited and divided into two groups: a Mebendazole-treated group and placebo group. The mebendazole and placebo groups were matched for age, sex, and complete blood count (CBC) with differential and liver and kidney function tests at baseline. On the third day, the C-reactive protein (CRP) levels were lower (2.03 ± 1.45 vs. 5.45 ± 3.95, *p* < 0.001) and the cycle threshold (CT) levels were higher (27.21 ± 3.81 vs. 24.40 ± 3.09, *p* = 0.046) significantly in the mebendazole group than in the placebo group on the third day. Furthermore, CRP decreased and CT dramatically increased on day three compared to the baseline day in the mebendazole group (*p* < 0.001 and *p* = 0.008, respectively). There was a significant inverse correlation between lymphocytes and CT levels in the mebendazole group (*r* = −0.491, *p* = 0.039) but not in the placebo group (*r* = 0.051, *p* = 0.888). Mebendazole therapy increased innate immunity and returned inflammation to normal levels in COVID-19 outpatients faster than it did in the placebo group in this clinical trial. Our findings add to the growing body of research on the clinical and microbiological benefits of repurposing antiparasitic therapy, specifically mebendazole, for SARS-CoV-2 infection and other viral infections.

## 1. Introduction

With the emergence and spread in 2019, Novel coronavirus (2019-nCoV) or severe acute respiratory syndrome coronavirus 2 (SARS-CoV-2) is a new global public health crisis [1]. Coronavirus disease 2019 (COVID-19) was reported as a cluster of illness in China in December 2019; it has since spread to nearly all continents, prompting the World Health Organization (WHO) to declare it a pandemic [2]. The first cases of COVID-19 in the Middle East and North Africa (MENA) region were reported in Iran in February 2020. By means of economic ties and religious tourism, these cases rapidly spread to neighboring Gulf states. Yemen was the last country in the Arab world to report its first case in April 2020. Most nations responded swiftly to the outbreak by instituting stringent border controls [3]. To date, more than 539 million cases of coronavirus and over 6 million deaths have been reported worldwide [4].

Jordan, a country with an upper-middle income, was severely affected by two waves of COVID-19, the first of which occurred in November 2020 and the second occurred in mid-to-late March 2021. Since then, the death toll in Jordan has skyrocketed to 14,000, and more than 1.6 million cases of COVID-19 have been diagnosed [4,5]. The increase in mortality caused by the pandemic and its control efforts could account for the excess deaths [6]. The most frequently reported symptoms of COVID-19 in Jordan are dry cough, general malaise, and fever. Hospitalization duration is proportional to the severity of chronic COVID-19 symptoms [7]. On 20 May 2022, the WHO reported a high COVID-19 vaccination rate in Jordan, with a total of 9,938,176 vaccine doses administered [5]. COVID-19 remains a fatal, highly pathogenic virus despite this [8]. The progression of mild COVID-19 to a more severe form may be prevented through early treatment [9].

As far as we are aware, only a handful of reports have raised concerns about the efficacy of medications used to treat COVID-19 patients. For instance, favipiravir was used with promising results in patients with mild to moderate COVID-19 infection [10,11]. Diverse health regulators and agencies have identified favipiravir as a potential treatment for COVID-19 in various regimen protocols [12,13]. However, its safety was minimal, as evidenced by significant elevations in liver enzyme levels, including aspartate aminotransferase and alanine transaminase, in the favipiravir group more frequently than in the placebo group [14]. In addition, ivermectin has been suggested as a potential treatment for COVID-19, but further research is required to determine whether this drug can benefit COVID-19 patients [15].

Mebendazole, a methyl-5-benzoylbenzimidazole-2-carbamate, is a broad-spectrum anthelmintic drug of the benzimidazole class that is effective against a number of nematode and cestode species by reducing parasitic worm tubulins. Mebendazole, as a novel Ran inhibitor, might work well for patients who are susceptible to the deadly inflammatory storm brought on by viral infections such as coronavirus. Mebendazole also has the potential to be an excellent and effective treatment for the majority of immunological disorders and viral infections. Moreover, the cellular membrane compartments of the host cells, particularly the endoplasmic reticulum, are intimately connected to the replication of positive-strand RNA viruses, such as the coronavirus family (ER). Besides that, coronaviruses are known to inhibit the host’s innate immune response by impairing the generation of interferon and may cause severe lung injury (ALI). Hence, preventing viral replication by a specific Ran inhibitor, producing more interferon, and suppressing cytokine storm target genes are the main goals of a potential treatment for viruses [16,17]. Therefore, the use of mebendazole in COVID-19 patients was linked to shorter hospital stays in the inpatient cohort and shorter times for symptom resolution in the outpatient cohort, as shown in a recent observational study [18].

Over the past four decades, mebendazole has been used to treat infectious parasites in humans. The FDA approved it for the treatment of gastrointestinal infections caused by Necator americanus or Ancylostoma duodenale (hookworm), Ascaris lumbricoides (ringworm), Enterobius vermicularis (pinworm), and Trichuris trichiura (worm) as a single or mixed parasitic infection in patients older than 2 years [19]. In addition, mebendazole is a new anti-proliferative agent that targets therapy-resistant cells. Mebendazole possesses cytotoxic properties, interacts synergistically with ionizing radiation and numerous chemotherapeutic agents, and stimulates antitumor immune responses [20]. Recent studies have demonstrated the effectiveness of mebendazole in treating a variety of cancers, including ovarian cancer [21], prostate cancer [22], brain tumors [23], acute myeloid leukemia [24], breast cancer [25], and colorectal cancer [26].

Mebendazole has a number of advantages, such as being well tolerated, inexpensive, and widely employed [27,28]. We chose to begin our investigation with mebendazole as an anti-SARS-CoV-2 drug because it possesses a number of desirable properties. For instance, mebendazole has an immuno-modulating effect by inducing interferon levels, and it is potent in upregulation for the pro-inflammatory M1-phenotype genes encoding cytokines [29]. Furthermore, mebendazole has been shown to activate the Mitogen-Activated Protein Kinase–Extracellular Signal-Regulated Kinase (MEK–ERK) pathway, with an advantage over other interferon inducers in that it restores the cell home [30]. Additionally, mebendazole has the ability to induce apoptosis via the dysregulation of Poly (ADP-ribose) Polymerase (PARP) and the activation of the cytoplasmic DNA sensor (cGAS) [31].

In addition to albendazole and oxibendazole, mebendazole may inhibit COVID-19 viral trafficking by impairing cellular microtubule integrity [32]. It has been demonstrated that mebendazole is capable of enhancing innate immune responses and is effective against SARS-CoV-2 [33]. The purpose of the current double-blind, randomized, placebo-controlled, phase II Clinical Trial was to evaluate the clinical efficacy, safety, and tolerability of the adjuvant use of mebendazole as a promising therapy for COVID-19 patients.

## 2. Results

### 2.1. Study Participants

One hundred and thirty-eight patients were initially screened and selected to participate in the study between 11 January and 12 March 2022. Due to the exclusion criteria, 69 patients received the assigned treatment and were enrolled in the study (34 in the treatment group and 35 in the placebo group). According to Table 1, the mebendazole and placebo groups were comparable with regard to gender (*p* > 0.05). Age, CBC with differential, and liver and kidney function tests at baseline were matched between both study groups (Table 2). Since subjects were recruited during the Omicron COVID-19 variant, thirty-two patients from the treatment group and thirty from the placebo group became PCR negative on day 5 and did not exhibit any symptoms. Therefore, we decided to compare all variables between the baseline day and the third day of drug administration.

### 2.2. Drug Safety

Seven patients in the drug group and two patients in the control group experienced adverse events, but none were considered serious according to the protocol definition. There was one case of diarrhea, four cases of nausea, one case of vomiting, one case of headache, one case of cough, one case of flu-like symptoms, and four cases of abdominal pain. Some of these adverse effects were only observed in the drug group and led to the study’s termination. Mebendazole-related gastrointestinal symptoms, including nausea, vomiting, and diarrhea, accounted for the majority of discontinuations. As shown in Table 3, there were no statistically significant differences between baseline day and day 3 for any safety measurements in the mebendazole group: alanine transaminase (ALT), aspartate transaminase (AST), urea, creatinine, sodium, and potassium (*p* value not significant between the two days).

### 2.3. Mebendazole Efficacy

**Figure 1:**
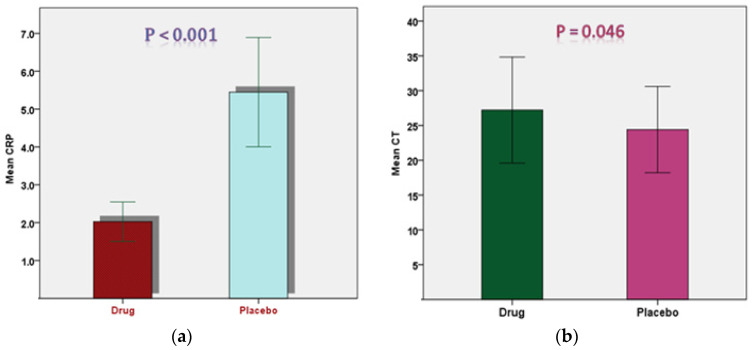
Comparison between the two study groups at day 3. CRP levels (**a**) and CT values (**b**) are significantly different between the mebendazole and placebo groups at the third day of the intervention (*p* < 0.001 and *p* = 0.046, respectively).

On the basis of primary and secondary clinical outcomes, the efficacy of mebendazole in COVID-19 patients was compared to that of a placebo drug. The primary endpoints of this study were the time from treatment initiation to negative PCR, the increase in PCR cycle threshold (CT), and the differential changes in WBC over the study timeframes. Changes between baseline and day 3 in C-reactive protein levels were the secondary efficacy endpoint. Table 4 revealed that there was no statistically significant difference between the baseline CRP and CT levels of the drug group and the control group. Third-day comparisons of CRP and CT levels between the two groups revealed significant differences, with lower CRP (2.03 ± 1.45 vs. 5.45 ± 3.95, *p* < 0.001) and higher CT levels (27.21 ± 3.81 vs. 24.40 ± 3.09, *p* = 0.046) in the mebendazole group than in the placebo group (Figure 1).

### 2.4. Outcomes Comparison between the Groups of the Study in the Two Timeframes

**Figure 2:**
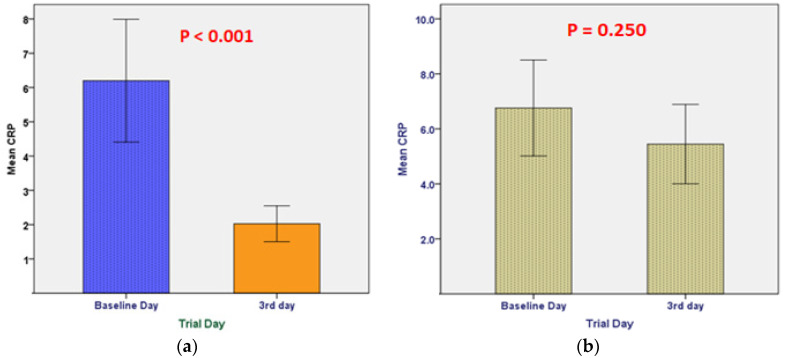
Comparison of CRP levels between the two days in the drug and placebo groups: CRP levels were significantly decreased on the third day as compared to baseline levels in the mebendazole group (**a**) but not in the placebo group (**b**), with *p*-values of <0.001 and 0.250, respectively.

**Figure 3:**
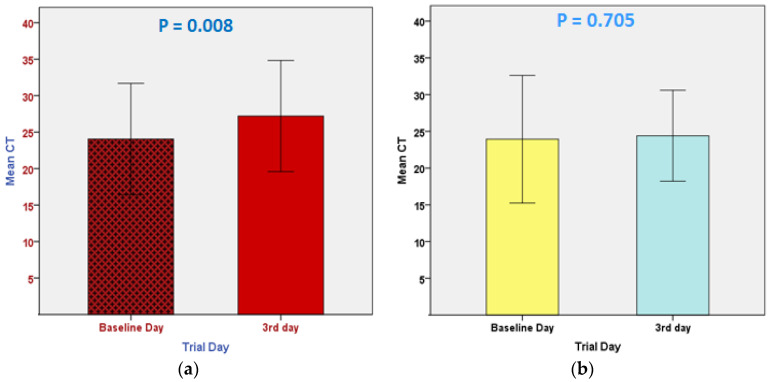
(**a**) The mean values of CT were significantly higher in the mebendazole group (*p* = 0.008). (**b**) An insignificant difference in CT values (*p* = 0.705) between the third day and baseline day in the placebo group.

**Figure 4:**
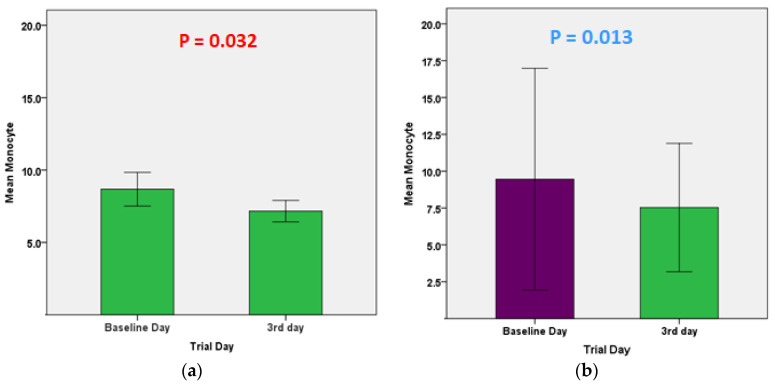
Monocytes were significantly reduced in the mebendazole (**a**) and placebo (**b**) groups on the third day of the trial (*p* = 0.032 and *p* = 0.013, respectively).

As shown in Figure 2, there was no statistically significant difference in CRP levels between day three and the baseline day in the control group (*p* = 0.25), whereas in the treatment group, there was a statistically significant difference between day three and the baseline day, with a dramatic decrease in CRP on day three compared to the baseline day (*p* < 0.001). In Figure 3, the CT levels increased significantly on the third day compared to the baseline day in the mebendazole group (*p* = 0.008) but not in the placebo group (*p* = 0.70). As shown in Figure 4, for all variables except for the monocyte count, there was no statistically significant difference between day 3 and the baseline in both study groups for the other primary endpoints, which include CBC with differential measures.

### 2.5. Relationship of Different Parameters in the Drug and Placebo Groups

Various parameters in the drug and placebo groups were subjected to a correlation analysis. The correlations between lymphocytes and CT levels in the study groups on the third day of the trial are shown in Table 5. In the mebendazole group, there was a significant inverse correlation between lymphocytes and CT levels (r = −0.491, *p* = 0.039), whereas no such correlation was observed in the placebo group (r = 0.051, *p* = 0.888).

## 3. Discussion

This randomized controlled trial was the first attempt to determine the safety and efficacy of mebendazole in COVID-19 outpatients. The results revealed a small number of adverse events comparable to those of other antiviral agents. Although mebendazole is often well tolerated, minor side effects have been noted, including nausea, vomiting, diarrhea, flatulence, and appetite loss. Seizures, convulsions, and hypersensitive reactions were severe and uncommon side effects that only a small number of patients had experienced [34]. Higher doses of mebendazole can cause neutropenia and thrombocytopenia [35]. Mebendazole’s safety as an anthelmintic is well established. Monitoring liver toxicity is among the most important adverse effects [36]. Long-term, high-dose (40 mg/kg per day) mebendazole usage was reported in the treatment of hydated echinococcosis in adults and children [37,38], with granulocytopenia, alopecia, pruritus, skin abscesses, and arthritis being the most common side effects [39,40]. In this clinical trial, mebendazole was well tolerated by COVID-19 patients with normal liver enzyme levels and renal function parameters at baseline and day 3. The daily dosage prescribed was 1000 mg, taken three times a day (six tablets). Such a large quantity of pills with substantial benefits may be preferable due to fewer difficulties in organizing drug administration and the possibility of adverse effects.

The efficacy of mebendazole in COVID-19 patients was evaluated using PCR cycle threshold (CT) elevation, CRP levels, and differential changes in WBC on day 3 between the drug group and the placebo group. On day 3, lower CRP and higher CT levels were observed in the mebendazole group but not in the control group; these changes were not observed on day zero in either group. In this double-blind, placebo-controlled, randomized trial, a positive PCR test was confirmed before the use of mebendazole at the start of COVID-19 symptoms. Several antiviral agents capable of managing COVID-19 hospitalizations have been investigated, including remdesivir, ritonavir, interferon, corticosteroids, cytokine storm blockers, and monoclonal antibodies, with similar results in mild to moderate COVID-19 patients [41,42,43,44,45,46,47]. Since no pharmacological agent has a well-established COVID-19 eradication effect that is effective, rapid, and inexpensive, it was crucial to evaluate a new potential anthelmintic agents with antiviral properties such as mebendazole in a prospective setting [48,49,50,51].

Mebendazole was discovered to be effective against a number of viruses such as HSV-1 and Zika virus [52]. Mebendazole, atovaquone, and ouabain were FDA-approved SARS-CoV-2 antivirals [48]. Atovaquone and mebendazole were the best SARS-CoV-2 drugs based on IC50, therapeutic plasma levels, pharmacokinetics, and side effects. Mebendazole inhibits SARS-CoV-2 replication by targeting Mpro, while atovaquone inhibits host purine metabolism [53]. Additionally, other drugs that kill parasites have been shown to kill viruses, especially in lab tests conducted in vitro. Ivermectin, for example, has been shown to have antiviral and immunomodulatory effects, which supports the idea that it could be used to treat COVID-19 disease [54].

Changes in the primary clinical endpoints (CT and WBC with differential) suggest a faster viral clearance in the mebendazole group than in the placebo group. In addition, the reduction and normalization of monocytes may indicate a more rapid eradication of the infection and its associated inflammation [55,56]. As for the secondary clinical endpoint (CRP), which indicates the inflammatory status of the body, the treatment group demonstrated a more rapid normalization of the elevated CRP levels, which favors a low incidence of COVID-19 complications [57]. To the best of our knowledge, this is the first double-blind, placebo-controlled, randomized trial to evaluate the efficacy of mebendazole in the treatment of outpatients with COVID-19.

In this clinical trial, mebendazole therapy in COVID-19 outpatients increased innate immunity and returned inflammation to normal levels more quickly than it did in the placebo group. Our findings add to the expanding corpus of research on the benefits of repurposing antiparasitic medication for both therapeutic and microbiological purposes, specifically mebendazole, for SARS-CoV-2 infection and other viral infections. The evidence for mebendazole’s antiviral effect comes from in vitro and in silico studies, and these are the first human data. As a result of the appearance of multiple COVID-19 variants with drastically different clinical manifestations, the significance of the repurposed agents varies with each COVID-19 variant and the demographic response of the exposed population. The low number of outpatients studied was a factor that future studies will take into consideration. This is because the enrollment process for the current study was impacted by the patients’ conditions, the variable epidemiology of COVID-19, and the participants’ transition from in-patients to outpatients during the study period. Future clinical trials with a larger sample size and an improved pharmaceutical formulation of mebendazole are strongly recommended for the treatment of autoimmune diseases and other viral infections, both in conjunction with existing standard treatments and other candidate repurposing agents.

## 4. Materials and Methods

### 4.1. Study Participants

Subjects were recruited from the emergency room of Amman Field Hospital, Jordan, as symptomatic COVID-19 patients with a positive PCR test and sent home for treatment. This double-blind, randomized controlled trial was sponsored by Al-Ahliyya Amman University, Amman, Jordan, and conducted from January to March 2022 at the emergency room of the Amman Field Hospital in Amman, Jordan. The study was conducted in accordance with the Declaration of Helsinki and its amendments, as well as the Guidelines for Good Clinical Practices issued by the European Union Committee for Medicinal Products (CPMP). The study protocol was reviewed and approved by the independent institutional review board (IRB) at Jordan Food and Drug Administration (JFDA), Jordan. This clinical trial study gained full approval from JFDA, with the approval number JPM-AAU-017 on 4 November 2021. All study participants provided their written informed consent. The work has been reported according to CONSORT (Consolidated Standards of Reporting Trials) guidelines [58].

### 4.2. Inclusion and Exclusion Criteria

Outpatients (*n* = 138) were screened and considered for participation in this trial. Due to the exclusion criteria, 69 patients received the assigned treatment and were enrolled in the study (34 in the Treatment group and 35 in the placebo group). The inclusion criteria were: (1) subjects with SARS-CoV-2 infection who did not require hospitalization; (2) subjects who were willing and able to provide written informed consent prior to enrollment in the study; and (3) subjects who had a PCR-positive test within 72 h prior to the consent form. The exclusion criteria were: (1) participants under the age of 18 years, (2) pregnant or nursing mothers, (3) patients with any liver abnormalities or current transaminases > three times the upper normal limit, (4) patients with any kidney abnormalities or current serum creatinine >1.5 mg/dL, (5) patients with known myopathy or elevated baseline creatinine kinase, (6) patients requiring sedation for mechanical ventilation, (7) patients requiring admission to the ICU, and (8) patients with an allergy to mebendazole or typical signs of hypersensitivity (rash, fever).

### 4.3. Blinding, Randomization, and Sample Size Calculation

This study was a randomized controlled trial with two arms consisting of placebo-controlled and drug-treated participants. To ensure data confidentiality, the generated randomization list was kept with the pharmacist responsible for blinding and drug distribution. To maintain blindness, the packaging and labeling of the drug and control treatments were identical. Participants, researchers, and study staff were unaware of the treatment assignments. A total of 58 patients (29 in the mebendazole group and 29 in the placebo control group) were required to obtain a mean effect size of 50% with an alpha error of 0.05 and a beta error not exceeding 0.15, which leaves the statistical power as 0.85.

### 4.4. Recruitment of Study Participants

COVID-19 outpatients were recruited and divided into two groups: the treatment group (Mebendazole with standard COVID-19 therapy) and the control group (with only standard COVID-19 therapy). In addition to the conventional treatment according to the Jordanian national protocol for the treatment of COVID-19 for ten days or until the first negative PCR, a matching placebo has been administered in accordance with the study’s randomization plan. The placebo was paired with a 1000 mg mebendazole (dose of two Vermox 500 mg tablets) three times daily. The standard care included all or some of the following COVID-19 repurposed medications: acetaminophen (500 mg), vitamin C (1000 mg twice/day), zinc (75–125 mg/day), vitamin D3 (5000 IU/day), azithromycin (250 mg/day for 5 days), levofloxacin (500 mg once orally for 5 days), desloratadine (5 mg once daily), and dexamethasone (6 mg/day).

### 4.5. Trial Procedure

Participants in the study were randomly assigned to receive Mebendazole 1000 mg (two tablets) three times daily for a total of ten days or until the first negative PCR. On the first day of drug administration, a research coordinator initiated daily phone calls for the duration of the study participation. The other objective of the follow-up was to assess patient adherence and the occurrence of adverse events. After signing the consent form and before starting the medication, each patient underwent home visits from the lab technician on days 3, 6, and 10 to collect blood samples for the following biochemical tests: COVID-19 PCR, liver function tests, kidney function tests, a complete blood cell count (CBC) with differential, C-reactive protein, and mebendazole plasma level.

### 4.6. Efficacy and Safety Assessment

This study’s primary endpoint was the time from treatment initiation to a PCR-negative result at day 3, as described in the procedures, as well as the changes in CBC with a differential panel over the study timeframes. The primary safety endpoint is the occurrence of any adverse event from the initial dose through the conclusion of the study, including any abnormalities in liver and kidney function tests measured on days 1 and 3. Changes in the C-reactive protein and PCR cycle threshold between days 1 and 3 represented the secondary efficacy endpoint.

### 4.7. Statistical Analysis

All data collection, processing, and analysis were performed using version 22.0 of IBM SPSS for Microsoft Windows (SPSS Inc., Chicago, IL, USA, version licensed to AAU). The Chi-square test was used to examine categorical (discrete) variable differences. Continuous data were presented as the mean ± standard deviation (SD) and subjected to the Shapiro–Wilk normality test; data that passed the normality test (followed a Gaussian distribution) were analyzed using parametric tests, while data that were not normally distributed were analyzed using nonparametric methods. For dependent data (outcomes before and after clinical intervention), paired student t-tests and Wilcoxon tests were used, whereas unpaired student *t*-tests and Mann–Whitney U tests were used for independent data (outcomes between the mebendazole group and the placebo group). Using Pearson’s correlation coefficient, a linear correlation was determined. Probability (*p*) values lower than 0.05 were considered statistically significant. *p* < 0.05 is considered statistically significant for mean differences. Utilizing G*Power 3.1, statistical power analyses and sample size calculations were conducted.

## Figures and Tables

**Table 1:** The study groups are matched for male and female numbers.

		Study Groups		*Chi*-Square	*p*-Value
		Drug	Placebo		
Gender	Female	21	19		
	Male	13	16	0.396	0.529
Total		34	35		

**Table 2:** Clinical characteristics of the study groups at baseline day.

	Mebendazole Group (*N* = 34)	Placebo Group (*N* = 35)	*p*-Value *
Age (years)	42.7 ± 13.3	38.9 ± 15.6	0.278
CRP (mg/L)	6.20 ± 5.22	6.76 ± 5.16	0.655
CT	24.07 ± 3.82	23.93 ± 4.34	0.894
Neutrophiles (%)	53.9 ± 13.2	48.5 ± 12.3	0.078
Lymphocytes (%)	34.59 ± 12.99	39.54 ± 12.91	0.117
Monocytes (%)	8.68 ± 3.39	9.46 ± 3.76	0.368
Urea (mg/dl)	23.27 ± 6.79	23.88 ± 11.97	0.793
Creatinine (mg/dl)	0.78 ± 0.13	0.79 ± 0.18	0.949
Sodium (mmol/dl)	139.8 ± 2.17	140.1 ± 1.9	0.564
Potassium (mmol/dl)	4.39 ± 0.46	4.46 ± 0.49	0.560
AST (U/L)	22.68 ± 11.26	25.34 ± 16.47	0.434
ALT (U/L)	25.56 ± 16.73	23.17 ± 11.33	0.492

Hematological and biochemical results for mebendazole and placebo groups at baseline day. All data are presented as the mean ± standard deviation. The study groups were matched for age. * The difference between the mebendazole and placebo groups was not significant (*p* > 0.05) in the percentages of neutrophiles, lymphocytes, and monocytes and biochemical parameters including C-reactive protein (CRP), cycle threshold (CT), aspartate aminotransferase (AST), alanine aminotransferase (ALT), and renal function tests.

**Table 3:** Safety of the mebendazole drug for the COVID-19 patients.

	Baseline Day (*N* = 34)	Day 3 (*N* = 31)	*p*-Value *
Renal Function			
Urea (mg/dL)	23.27 ± 6.79	25.13 ± 6.62	0.269
Creatinine (mg/dL)	0.78 ± 0.13	0.80 ± 0.15	0.575
Sodium (mmol/dL)	139.8 ± 2.2	135.5 ± 25.1	0.351
Potassium (mmol/dL)	4.39 ± 0.46	10.05 ± 25.10	0.219
Liver Function			
AST	22.68 ± 11.26	21.26 ± 11.10	0.611
ALT	25.56 ± 16.73	23.35 ± 11.78	0.539

All data are presented as the mean ± standard deviation. * No significant difference in the kidney and liver function tests between baseline day and day 3 in the mebendazole group. AST, aspartate aminotransferase; ALT, alanine aminotransferase.

**Table 4:** Comparison of CRP and CT levels between the two groups at baseline day.

Parameter	Study Groups	N	Mean ± SD	*p*-Value *
CRP (mg/L)	Drug	34	6.20 ± 5.22	0.655
	Placebo	35	6.76 ± 5.16	
CT	Drug	34	24.06 ± 3.82	0.984
	Placebo	35	23.93 ± 4.34	

* No significant difference was observed between the mebendazole and placebo groups at baseline day. CRP, C-reactive protein; CT, cycle threshold; SD, standard deviation.

**Table 5:** Correlation analysis between lymphocytes and CT levels in the two study groups at day 3 of the trial.

Study Groups	Lymphocytes vs. CT	
	Pearson’s Correlation Coefficient (r)	*p*-Value
Treatment group	−0.491	0.039 *
Placebo group	0.051	0.888

* Lymphocytes and CT values are significantly and positively correlated at day 3 only in the treatment group.

## Data Availability

Data is contained within the article.
